# Screening of potential biomarkers in peripheral blood of patients with depression based on weighted gene co-expression network analysis and machine learning algorithms

**DOI:** 10.3389/fpsyt.2022.1009911

**Published:** 2022-10-17

**Authors:** Zhe Wang, Zhe Meng, Che Chen

**Affiliations:** School of Chinese Medicine, Ningxia Medical University, Yinchuan, China

**Keywords:** biomarkers, weighted correlation network analysis, machine learning, depression, blood

## Abstract

**Background:**

The prevalence of depression has been increasing worldwide in recent years, posing a heavy burden on patients and society. However, the diagnostic and therapeutic tools available for this disease are inadequate. Therefore, this research focused on the identification of potential biomarkers in the peripheral blood of patients with depression.

**Methods:**

The expression dataset GSE98793 of depression was provided by the Gene Expression Omnibus (GEO) (https://www.ncbi.nlm.nih.gov/gds). Initially, differentially expressed genes (DEGs) were detected in GSE98793. Subsequently, the most relevant modules for depression were screened according to weighted gene co-expression network analysis (WGCNA). Finally, the identified DEGs were mapped to the WGCNA module genes to obtain the intersection genes. In addition, Gene Ontology (GO), Disease Ontology (DO), and Kyoto Encyclopedia of Genes and Genomes (KEGG) functional enrichment analyses were conducted on these genes. Moreover, biomarker screening was carried out by protein-protein interaction (PPI) network construction of intersection genes on the basis of various machine learning algorithms. Furthermore, the gene set enrichment analysis (GSEA), immune function analysis, transcription factor (TF) analysis, and the prediction of the regulatory mechanism were collectively performed on the identified biomarkers. In addition, we also estimated the clinical diagnostic ability of the obtained biomarkers, and performed Mfuzz expression pattern clustering and functional enrichment of the most potential biomarkers to explore their regulatory mechanisms. Finally, we also perform biomarker-related drug prediction.

**Results:**

Differential analysis was used for obtaining a total of 550 DEGs and WGCNA for obtaining 1,194 significant genes. Intersection analysis of the two yielded 140 intersection genes. Biological functional analysis indicated that these genes had a major role in inflammation-related bacterial infection pathways and cardiovascular diseases such as atherosclerosis. Subsequently, the genes S100A12, SERPINB2, TIGIT, GRB10, and LHFPL2 in peripheral serum were identified as depression biomarkers by using machine learning algorithms. Among them, S100A12 is the most valuable biomarker for clinical diagnosis. Finally, antidepressants, including disodium selenite and eplerenone, were predicted.

**Conclusion:**

The genes S100A12, TIGIT, SERPINB2, GRB10, and LHFPL2 in peripheral serum are viable diagnostic biomarkers for depression. and contribute to the diagnosis and prevention of depression in clinical practice.

## Introduction

Depression is a prevalent mental disorder that causes persistent depressed mood, retardation of thinking, and, in severe cases, leads to self-harm and suicidal behavior (1). To date, the global depression cases exceed 300 million, making it the largest single contributor to the global burden of diseases (2). Despite extensive academic research on the biology of depression, the pathophysiological mechanisms of depression remain elusive. Currently, the diagnosis and treatment of depression are still based on the subjective assessment of symptoms, with a lack of objective biomarkers (3, 4). In addition, most antidepressant drugs in clinical settings have low efficacy and extended dosage with multiple adverse effects, resulting in heavy physical, psychological, and economic burdens on patients. Therefore, the research and development of new diagnostic and therapeutic biomarkers for depression are necessary.

The remarkable advancement of sequencing technologies has enabled the widespread use of bioinformatics in several fields. In medical research, the most common use of sequencing technologies is to identify potential biomarkers in tumor and non-tumor diseases to assist physicians in predicting patient prognosis and response to therapy (5, 6). For example, weighted correlation network analysis (WGCNA, also called weighted gene co-expression network analysis) combined with random forest models identified multiple pathological processes and associated featured genes such as hormone secretion regulation, airway remodeling, and immune response regulation, which can help in determining the progression of asthma (7). Furthermore, WGCNA combined with the random forest model, SVM–REF model, and LASSO analysis identified TMCC2, GYPA, and BPGM as peripheral serum biomarkers in patients with steroid-induced femoral head necrosis (8). However, most bioinformatics studies for depression did not involve WGCNA + ML.

Therefore, the current study integrated WGCNA and the three machine learning algorithms, including the random forest model, SVM–REF model, and LASSO analysis, to identify potential biomarkers in the peripheral blood of patients suffering from depression. First, disease transcription data were downloaded from the GEO database for DEG analysis and WGCNA hub module gene analysis. Intersection genes between the two were considered depression-related key targets and were subjected to functional analysis. Subsequently, potential biomarkers were identified by the three aforementioned machine learning algorithms for the investigation of the functions and regulatory mechanisms of these genes. Overall, the outcomes of this research may help us understand the pathogenesis of depression in further detail and assist in its diagnosis and treatment.

## Materials and methods

### Data sources

Gene expression files for depression were collected from the GSE98793 dataset in the Gene Expression Omnibus (GEO)^1^ database. After excluding 64 patients with depression accompanied by anxiety, expression data of 64 depressed people alone and 64 healthy controls was retained. In addition, including the GSE76826 dataset (10 depressed patients and 12 healthy controls) and the GSE201332 dataset (20 depressed patients and 20 healthy controls), the expressions of the three datasets are all derived from human blood tissue.

### Identification and functional enrichment analysis of differentially expressed genes in the peripheral blood of patients with depression

The “limma” and “GSEABase” packages in R (4.2.0) software were used for the identification of GEGs and GSEA functional analysis. The screening criteria for DEGs between the depression group and the control group were *P*-value < 0.05 and |LogFC| > 0.2.

### Screening of target genes by weighted gene co-expression network analysis

The WGCNA was utilized for constructing unsigned co-expression networks for the identification of co-expression modules. Initially, samples were examined for missing values and then clustered. Afterward, a “soft” threshold power (β) was estimated according to the criteria of scale-free topology to construct a biologically important scale-free network. Moreover, a topological overlap matrix (TOM) was devised on the basis of an adjacency matrix, and a dynamic tree-cutting algorithm was used for detecting gene modules. In addition, we calculated gene significance (GS), module membership (MM), and associated modules with clinical features, and then the network of feature genes was visualized. Finally, we took the intersection between DEGs and WGCNA-derived significant module genes to obtain potential gene targets of depression.

### Gene ontology, disease ontology, and kyoto encyclopedia of genes and genomes enrichment analyses

Gene Ontology (GO), Disease Ontology (DO), and Kyoto Encyclopedia of Genes and Genomes (KEGG) enrichment analyses of potential targets were carried out by setting a *P*-value < 0.05 as a filtering criterion for functional analysis using the “clusterProfiler” package. Afterward, GO analysis was carried out to identify hub gene-related biological processes (BPs), molecular functions (MFs), and cellular components (CCs). DO analysis was done for the identification of diseases with frequent involvement of the aforementioned genes. In addition, KEGG enrichment analysis was carried out to find the signaling pathways enriched by the potential targets.

### Protein-protein interaction network construction

The STRING^2^ database, with a minimum required interaction score set to medium confidence (0.4), was used to construct an interaction network of potential targets, and Cytoscape software was utilized to see the results. Finally, we considered the genes included in the network as hub genes in the pathological process of depression and selected them for subsequent biomarker screening in peripheral serum of patients with depression.

### Blood marker screening in patients with depression by machine learning algorithms

Three machine learning algorithms were used for this study. First, the “e1071” package was used for performing the SVM-REF analysis. Subsequently, the “randomForest” package was used for performing the random forest analysis, and the ten most important genes were retained. Furthermore, the “glmnet” package was used for performing LASSO regression analysis. The intersection genes obtained as a result of the three analyses were considered potential blood biomarkers for patients with depression. Moreover, a hub gene-based nomogram was constructed using the “rms” package. Finally, we predicted the prevalence of depression in the population based on the expression of potential biomarkers.

### Correlation and gene set enrichment analyses of potential biomarkers

Correlation analysis of potential biomarker expression was performed using the “corrplot” package. Subsequently, the GSEA of the potential biomarkers was performed to further understand their possible functions.

### Expression of potential biomarkers

The Wilcoxon rank-sum test was used for analyzing the expression levels of potential biomarkers due to the large sample size in this study. First, the expression profiles of potential biomarkers in the GSE98793 dataset were measured. Subsequently, these profiles were validated in the GSE76826 dataset. In addition, GeneCards^3^ is a comprehensive database containing proteomics, genomics and transcriptomics functions (9). We screened the genes related to depression and verified the obtained biomarkers again.

### Immune infiltration analysis

A clinical study has revealed that the immune system is closely related to the pathophysiology of depression (10). Therefore, the “CIBERSORT.R” package was used for analyzing the expression status of 22 distinct immune cells in the tissues of patients. Subsequently, a correlation analysis between potential biomarkers and 22 immune cells was conducted to further understand the changes occurring in the immune system in patients with depression.

### Regulatory mechanisms of potential biomarkers

To further understand the potential biomarkers’ mechanism of action in the onset and progression of depression, an enrichment analysis of potential biomarker-related transcription factors (TFs) was performed based on the NetworkAnalyst^4^ platform (TF). In addition, biomarker regulation-related miRNAs were predicted.

### Potential drug screening

The development of novel therapeutic agents is necessary because a large number of available commercial depression drugs fail to achieve satisfactory efficacy. In this research, we identified biomarkers based on the previous study and searched Drug Signatures Database (DSigDB) in Enrichr^5^ website for potential biomarker-related drug prediction. Furthermore, the structures of the corresponding drugs were screened using the PubChem^6^ database and visualized.

### Prediction of clinical diagnostic ability of biomarkers

We performed receiver operating characteristic (ROC) analysis in two datasets, GSE98793 (source set) and GSE76826 (validation set), and calculated area under the curve (AUC) values through the “pROC” package to evaluate whether potential biomarkers are good Distinguish between depression samples and control samples. Finally, the GSE201332 dataset was used to explore the biomarkers with the most clinical diagnostic value.

### Mfuzz expression pattern clustering and functional analysis of optimal diagnostic biomarkers

In the study, we used the “Mfuzz” package to cluster Mfuzz expression patterns according to the expression levels of the optimal diagnostic biomarkers, and calculated the ssGSEA (single sample Gene Set Enrichment Analysis) scores of different clustering modules as well as in the depression group. and the expression characteristics between the normal group, and then calculate the correlation between the clustering module and the biomarker, and finally, obtain the gene module most closely related to the biomarker. Subsequently, the gene modules we obtained were subjected to GO and KEGG analysis to explore the module gene functions. Finally, we intersected this module gene with the potential targets of depression obtained in this study to obtain the core genes associated with the optimal biomarkers in depression.

## Results

### Identification of differentially expressed genes in peripheral serum of depression patients

**FIGURE 1:**
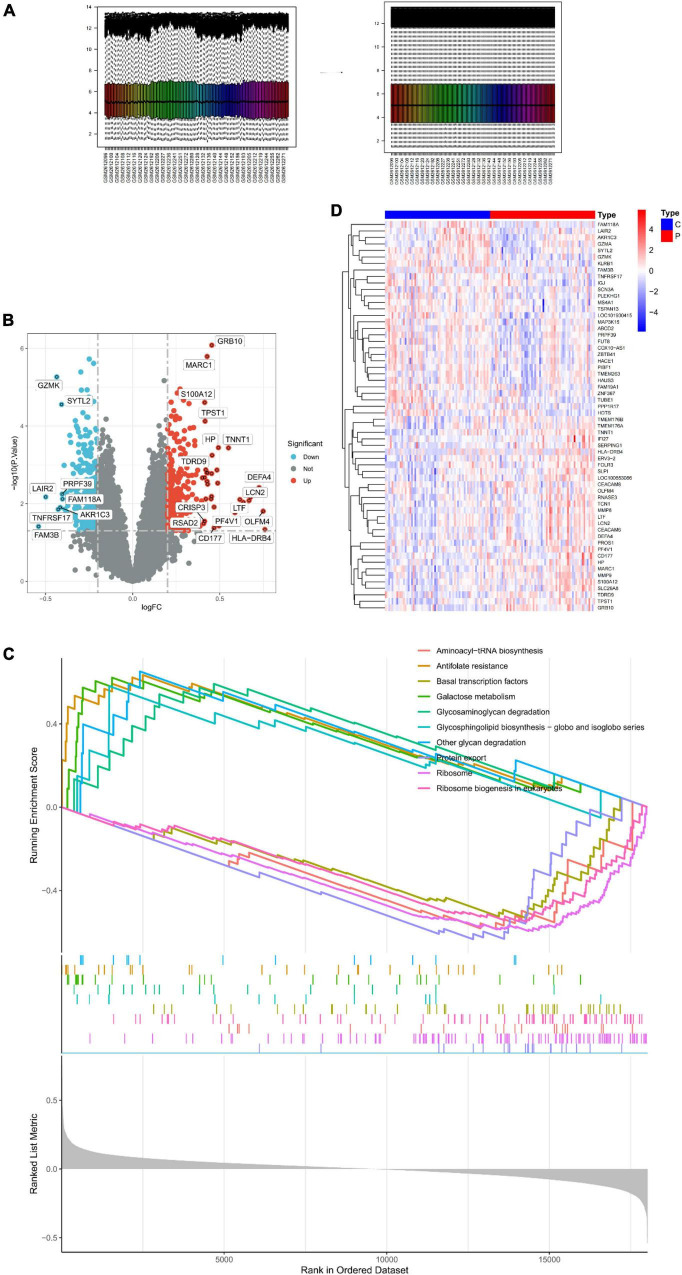
Identification of DEGs in peripheral serum of depression patients. **(A)** Sample normalization process. **(B)** Volcano Plot showing the expression characteristics of DEGs, where red represents gene upregulation in the depression group, and blue represents gene upregulation in the normal control group. **(C)** Heat map showing the expression of the top 30 DEGs in the sample. **(D)** GSEA of DEGs.

The samples were first standardized (Figure 1A) to obtain 550 DEGs in the peripheral serum of depression patients; 242 among these DEGs were up-regulated and 308 were down-regulated (Figure 1B). The expression of top 30 genes with the most significant differences between the depression and normal control groups was visualized in Figure 1C. Finally, GSEA of the 550 DEGs yielded the ribosome, protein export, and antifolate resistance pathways (Figure 1D).

### Hub gene screening using weighted gene co-expression network analysis

**FIGURE 2:**
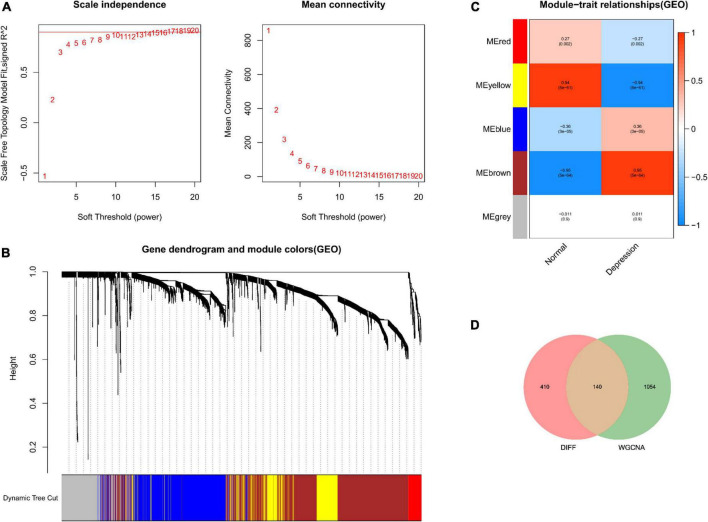
Depression-related hub module identification. **(A)** Left: Scale-free fit index (proportional independence, *y*-axis) as a function of soft threshold power (*x*-axis); right: mean connectivity (degrees, *y*-axis) as a function of soft threshold power (*x*-axis). **(B)** Gene dendrogram as a result of clustering, where the colored rows below the dendrogram denote the module assignment identified by dynamic tree shearing. **(C)** Module–feature correlations. Individual rows in the heat map correspond to an ME, whereas each column corresponds to a clinical feature. Individual cells contain the corresponding correlation coefficient and *P*-value. **(D)** DEGs mapped to WGCNA module genes.

No outliers were detected during sample clustering. Subsequently, the scale-free fit index was set to 0.9 to obtain a minimum soft threshold of 9 for constructing the scale-free network (Figure 2A). In addition, modules with an Eigen factor greater than 0.75 (the lowest number of genes in the module was set to 50) were combined to obtain four modules (Figure 2B). Module membership (MM) indicates the correlation of module gene expression values with module Eigen gene (ME). Gene significance (GS) indicates the correlation of module genes with samples. The modules were linked to the clinical characteristics by calculating MM and GS values. The brown module was most associated with depression (Figure 2C). Finally, the brown module genes were intersected with DEGs, yielding 140 depression-related potential targets (Figure 2D).

### Gene ontology, disease ontology, and kyoto encyclopedia of genes and genomes enrichment analyses

**FIGURE 3:**
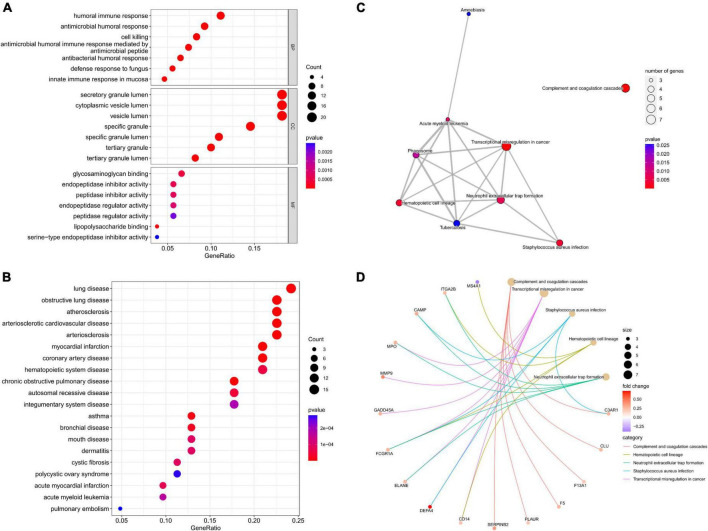
Functional analysis of potential targets. **(A)** Top 7 BPs, CCs, and MFs; **(B)** top 20 Dos; **(C)** all KEGG pathways; **(D)** top five KEGG pathways and enrichment genes.

Enrichment analyses were carried out to study the biological functions (BFs) of potential gene targets. GO analysis suggested that these targets exerted multiple functions such as lipopolysaccharide binding, glycosaminoglycan binding, and endopeptidase inhibitor activity. In addition, they were associated with many CCs such as specific granule, specific granule lumen, and secretory granule lumen, and were involved in several BPs such as antimicrobial humoral response, antimicrobial humoral response mediated by an antimicrobial peptide, and antibacterial humoral response (Figure 3A). KEGG enrichment analysis findings revealed that these target genes were primarily enriched by signaling pathways including the complement and coagulation cascades, transcriptional misregulation in cancer, *Staphylococcus aureus* infection, and hematopoietic cell lineage (Figures 3C,D). DO analysis revealed that the targets were closely associated with myocardial infarction, atherosclerosis, and arteriosclerotic cardiovascular disease (Figure 3B).

### Protein-protein interaction network construction

**FIGURE 4:**
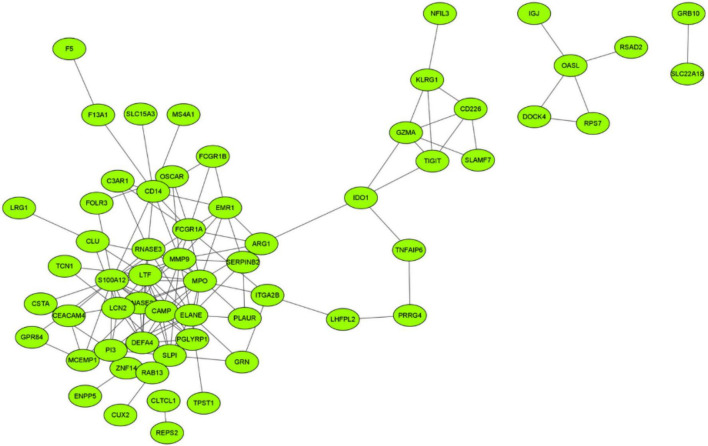
Interaction network of potential targets.

An interaction network of potential targets was constructed (Figure 4). The network consisted of 60 nodes and 133 lines. Several genes at the center of the network that affected other genes include S100A12, ELANE, MMP9, MPO, LTF, and FCGR1A. Furthermore, 60 node genes in this network were selected for subsequent biomarker screening.

### Biomarker screening in peripheral serum of depression patients based on machine learning algorithms

**FIGURE 5:**
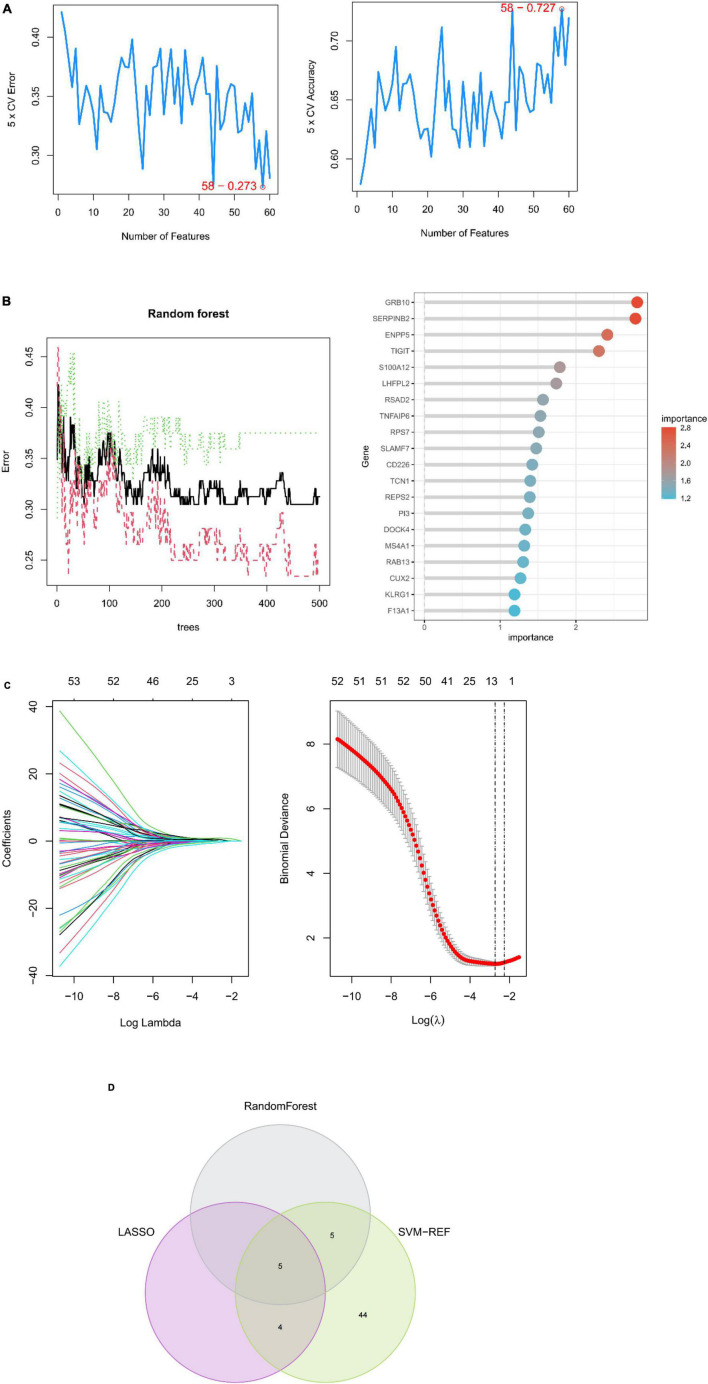
Machine learning-based potential biomarker screening. **(A)** SVM–REF model with the optimal error rate when the number of signature genes was 58. **(B)** RANDOM forest model and the top 20 genes in terms of importance. **(C)** LASSO regression model. **(D)** Biomarkers.

A machine learning approach was used in this study to further investigate depression-related potential biomarkers in 60 key targets. SVM–REF analysis revealed that the model incorporating 58 genes had the best accuracy (Figure 5A). The top 10 genes from random forest results were selected as candidate biomarkers (Figure 5B). In addition, the LASSO regression model was designed based on depression as well as control samples. The λ analysis highlighted that the model could accurately predict depression when λ = 10. Therefore, LASSO analysis yielded 10 candidate genes (Figure 5C). Finally, the results from the three algorithms were combined, yielding S100A12, TIGIT, SERPINB2, GRB10, and LHFPL2 in peripheral blood as depression-related potential biomarkers (Figure 5D).

**FIGURE 6:**
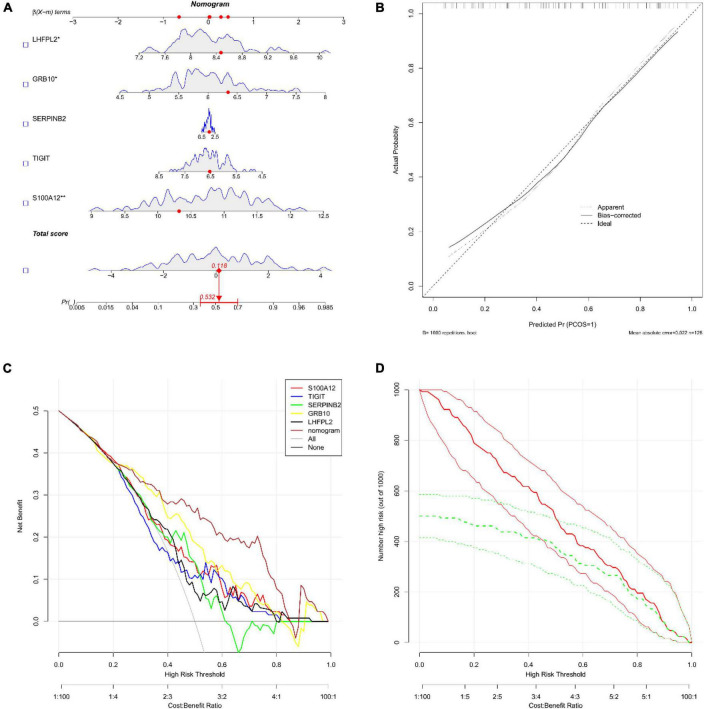
**(A)** Nomograms; we obtained the corresponding scores for each genetic variable, drew a vertical line above the “points” axis and summed the scores of all predictor variables and found the final value on the “total score” axis, and then drew a straight line on the “probability” axis to determine the patient’s risk of depression’s. **(B)** Calibration curve **(C)** decision curve analysis (DCA); the horizontal line indicated no depression, the gray diagonal line indicated no depression, and the column diagram showed more net benefit within the threshold probability range. **(D)** Clinical impact curves (CIC); red curves (the number of high-risk individuals) indicated the number of individuals classified as positive (high-risk) by the model at each threshold probability; the green curves (the number of high-risk individuals with outcomes) were the number of true positives at each threshold probability.

Subsequently, the diagnostic column line diagram showed that the expressions of five biomarkers assisted in the clinical diagnosis of depression, and we assessed the probability of a patient being diagnosed with depression by integrating the scores of each gene (Figure 6A), and the calibration curves also showed that the column line diagram model had good predictive ability (Figure 6B). In addition, DCA (Figure 6C) and CIC (Figure 6D) intuitively demonstrated that columnar diagrams have a superior overall net benefit over a wide and practical range of threshold probabilities, suggesting that the columnar diagrams obtained in our current study can help clinicians more accurately assess the prognosis of patients. The above results showed that S100A12, TIGIT, SERPINB2, GRB10, and LHFPL2 all have the potential to be diagnostic biomarkers in depression.

### Correlation between potential biomarkers and gene set enrichment analysis

**FIGURE 7:**
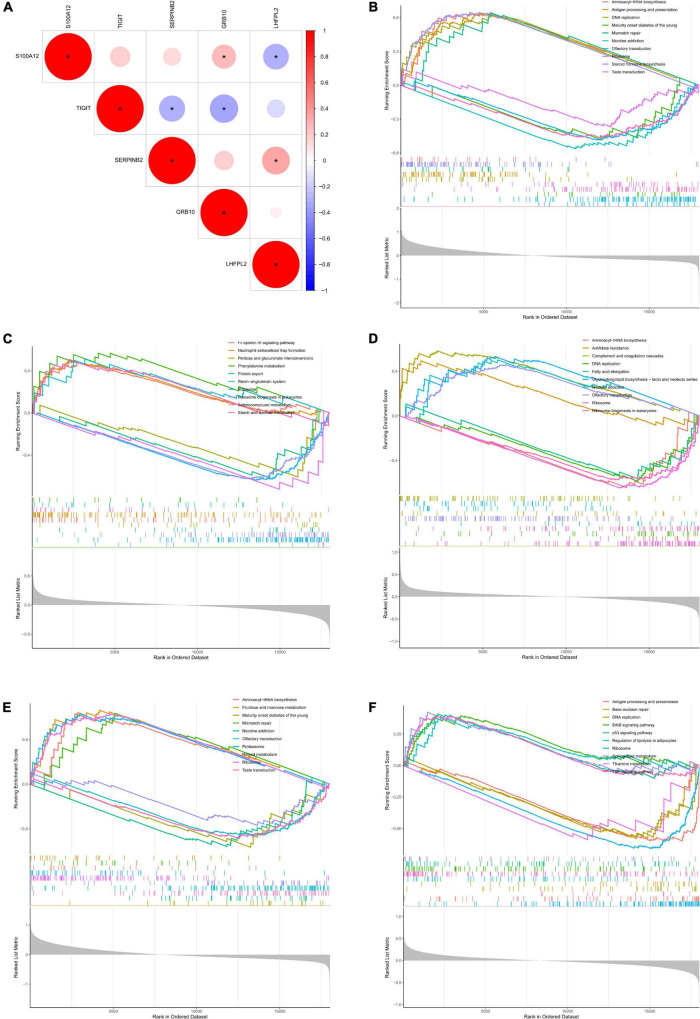
**(A)** Correlation analysis between potential biomarkers. **(B–F)** GSEA functional analysis of potential biomarkers (**B**, TIGIT, **C**, GRB10, **D**, LHFPL2, **E**, S100A12, **F**, SERPINB2).

Initially, the correlation analysis showed that TIGIT had a significantly negative correlation with the level of SERPINB2 and GRB10 expression in patients with depression. In addition, S100A12 showed a significant negative correlation with LHFPL2 expression level and a significant positive correlation with GRB10 expression level (Figure 7A). Subsequently, we performed GSEA functional analysis of the five potential biomarkers and detected multiple pathways including ribosome function, ribosome biogenesis in eukaryotes, protein export, and antifolate resistance, which were consistent with the DEG functional analysis results. In addition, pro-inflammatory cytokines and immune cell pathways such as IL-17, TNF, B cell receptor, and T cell receptor signaling pathway were identified, indicating that inflammation plays a key role in the onset and development of depression. Finally, the results of the GSEA functional analysis of the five potential biomarkers were visualized as shown in Figures 7B–F.

### Immune cell infiltration analysis

**FIGURE 8:**
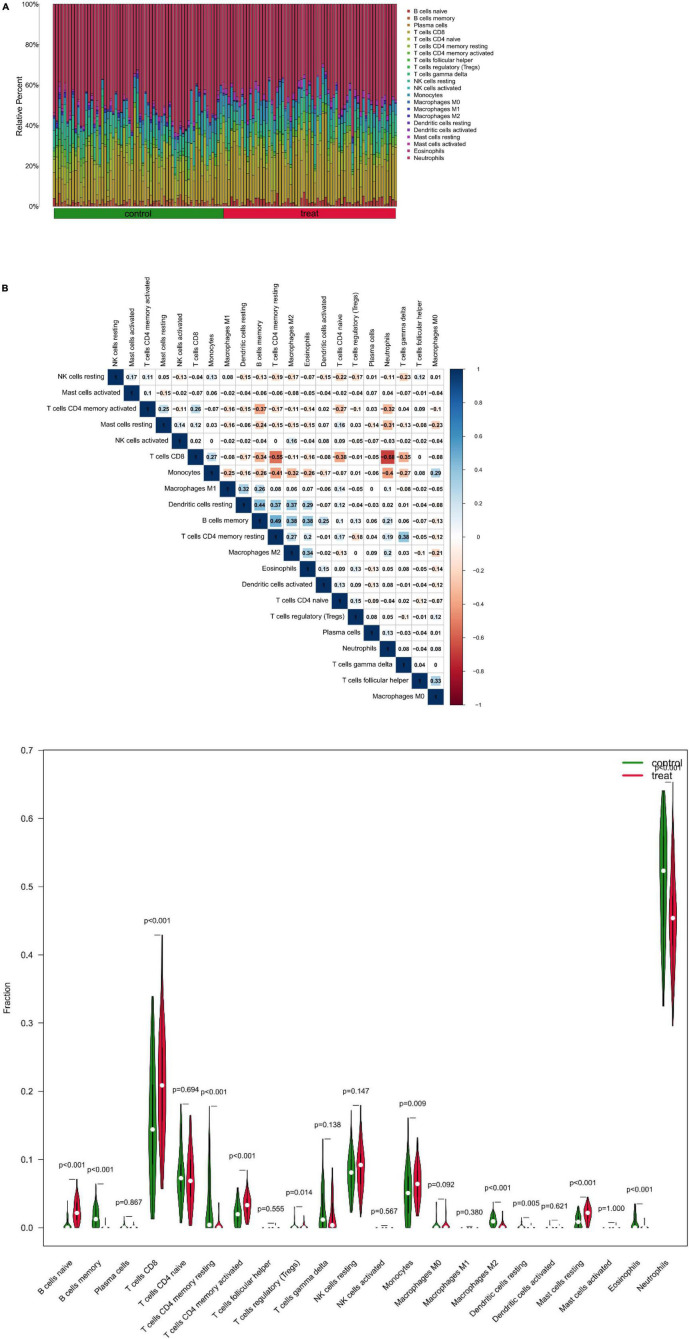
**(A)** Relative proportion of peripheral blood infiltrates of 22 distinct subtypes of immune cells in patients with depression. **(B)** Correlation between 22 distinct populations of immune cells, where red and blue denote positive and negative correlations, respectively, and white denotes no correlation between the designated immune cell populations. **(C)** comparison of 22 immune cell types, where green and red denote the normal and depression groups, respectively. Control represents the normal group, treat represents the depression group.

At first, the infiltration status of 22 different types of immune cells in the serum of patients with depression was measured (Figure 8A). Subsequently, the correlation between these cell populations was assessed (Figure 8B). A strong negative correlation between T cells CD8 and neutrophils (*r* = –0.68) and a stronger positive correlation between B cells memory and T cells CD4 memory resting (*r* = 0.49) was observed. In addition, the immune cells’ infiltration levels in peripheral blood between the depression and normal control groups were compared. Immune cells such as B cells naive, T cells CD8, and mast cells resting had a significantly higher expression in patients in the depression group, whereas B cells memory and T cells CD4 memory resting showed a significantly higher expression in patients in the normal control group (Figure 8C).

**FIGURE 9:**
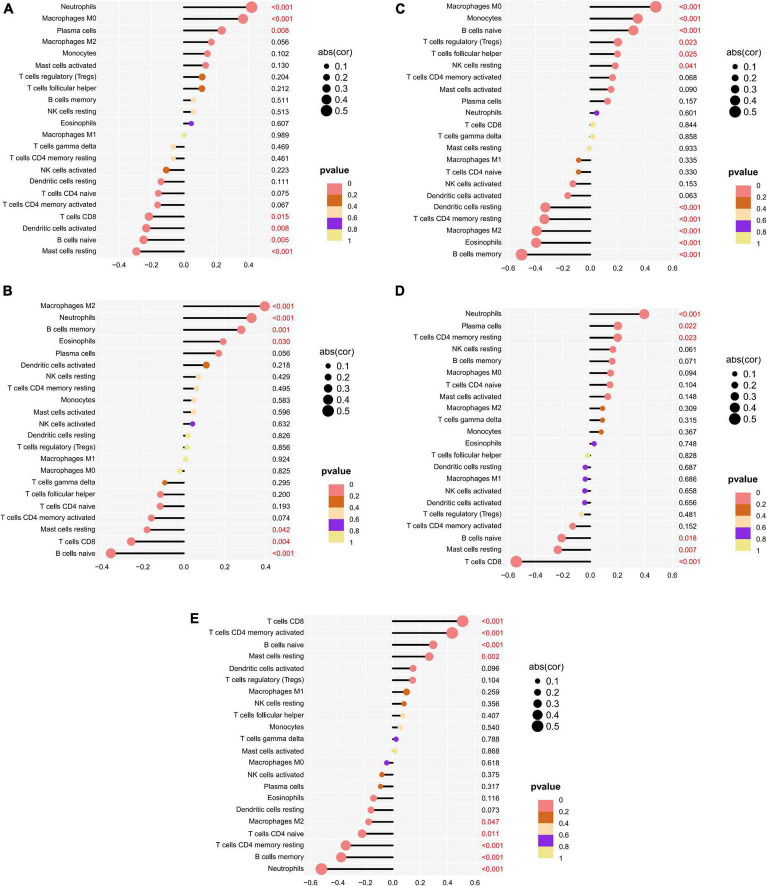
Relationship between potential biomarkers and immune infiltration in depression. **(A)** GRB10, **(B)** LHFPL2, **(C)** S100A12, **(D)** SERPINB2, **(E)** TIGIT.

The correlation of five potential biomarkers with immune cell infiltration was explored (Figure 9). GRB10 expression showed a positive link to the infiltration status of neutrophils, macrophages MO, and plasma cells, and a negative correlation with the infiltration status of B cells naive and resting mast cells. lHFPL2 expression showed a positive link to the infiltration status of neutrophils, macrophages M2 and B cells memory, and negatively correlated with B cells naive and T cells CD8. Moreover, S100A12 expression was positively linked to the infiltration status of macrophages MO, monocytes, and naïve B cells and negative correlation with the infiltration status of eosinophils, B cells memory, and macrophages M2. SERPINB2 expression had a positive link to the infiltration status of plasma cells, neutrophils, and T cells CD4 memory resting, and a negative correlation with the infiltration status of T cells CD8, mast cells resting, and B cells naive. Furthermore, TIGIT expression had a positive link to the infiltration status of T cells CD8, T cells CD4 memory activated, and B cells naive and a negative correlation with the infiltration status of neutrophils, B cells memory, and T cells CD4 memory resting.

### Expression characteristics of potential biomarkers

**FIGURE 10:**
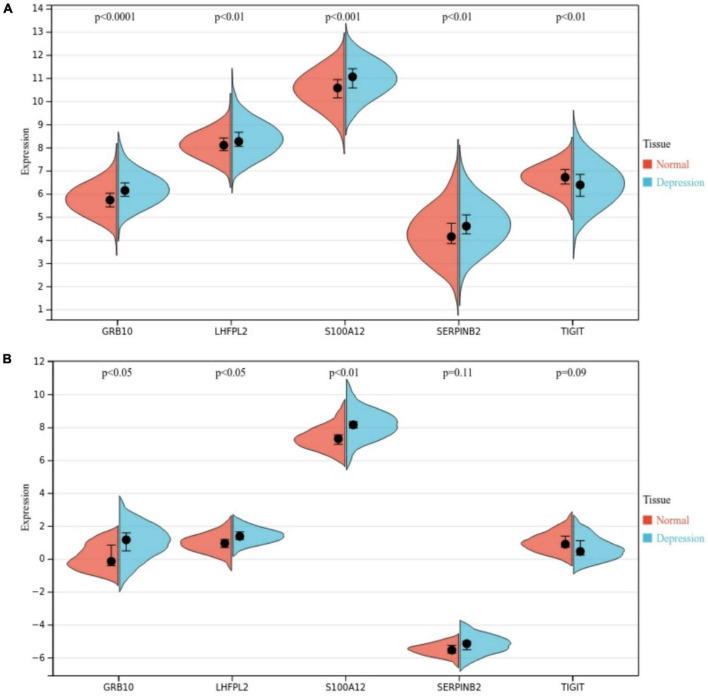
**(A)** Potential biomarker expression in the GSE98793 dataset. **(B)** Potential biomarker expression in the GSE76826 dataset.

We further investigated the role of S100A12, TIGIT, SERPINB2, GRB10, and LHFPL2 in depression and observed their expression profiles in patients. The expression level of 100A12, SERPINB2, GRB10, and LHFPL2 was higher in the depression group, whereas TIGIT showed a high expression profile in the normal control group (Figure 10A). The above findings were also validated in the GSE76826 dataset (Figure 10B). Moreover, our search for depression-related genes in the Genencards database also confirmed that the above biomarkers were strongly associated with depression. Among them, the relevance scores of S100A12, TIGIT, SERPINB2, GRB10, and LHFPL2 with depression were 4.356, 4.232, 3.064, 1.516, and 0.698, respectively.

### Regulatory mechanisms of potential biomarkers

**FIGURE 11:**
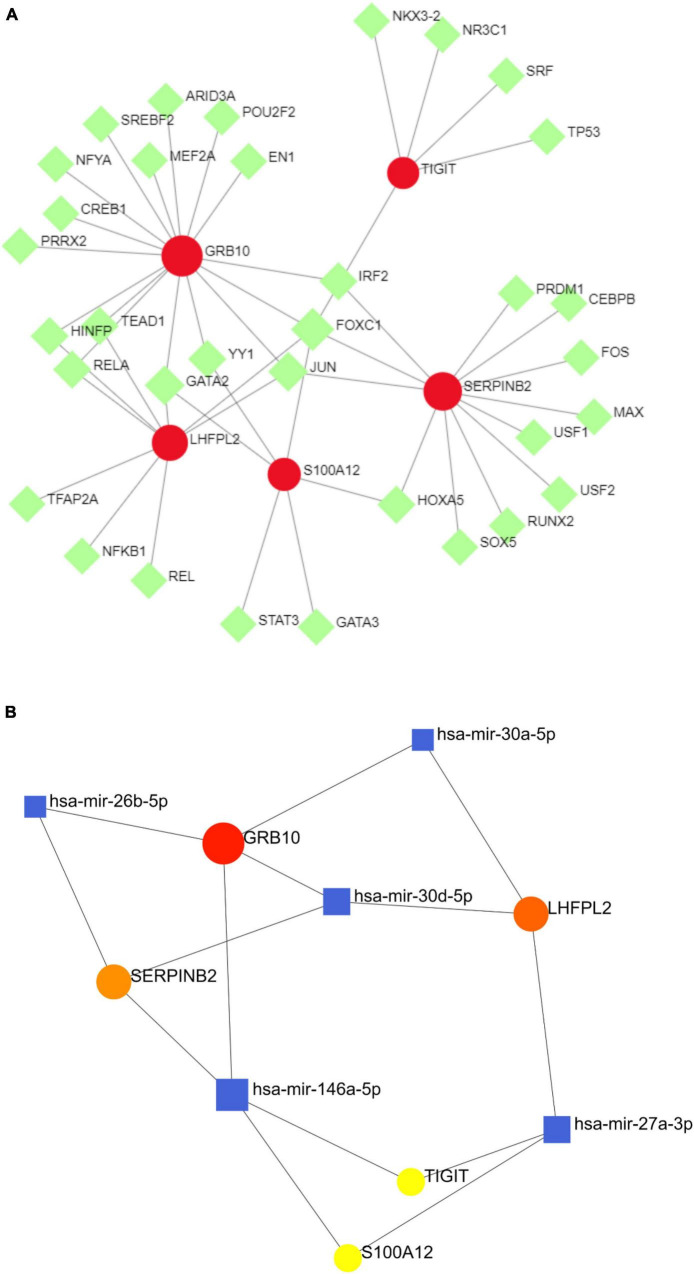
**(A)** TF–potential biomarker network. **(B)** miRNA–potential biomarker network, where the red nodes symbolize potential biomarkers, the green nodes symbolize TFs, and the blue nodes denote miRNAs.

To investigate the regulatory mechanisms of S100A12, TIGIT, SERPINB2, GRB10, and LHFPL2, their TFs were predicted, and a TF–potential biomarker network was constructed (Figure 11A). S100A12 may be regulated by STAT3, GATA3, and GATA2; TIGIT may be regulated by TP53, SRF, and NR3C; LHFPL2 may be regulated by TFAP2A, NFKB1, and REL; SERPINB2, and GRB10 may be regulated by JUN, FOXC1, and IRF2. Subsequently, a miRNA–potential biomarker network was constructed (Figure 11B) to obtain miRNAs, including has-mir-164a-5p, that play key regulatory roles.

### Prediction of therapeutic drugs

**FIGURE 12:**
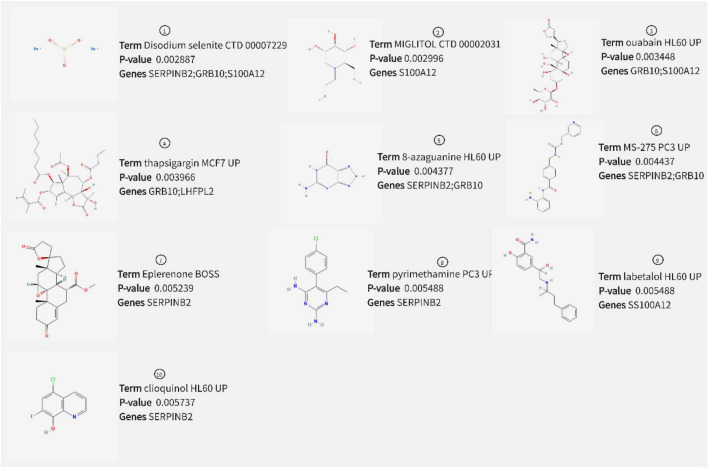
Drug prediction results.

**FIGURE 13:**
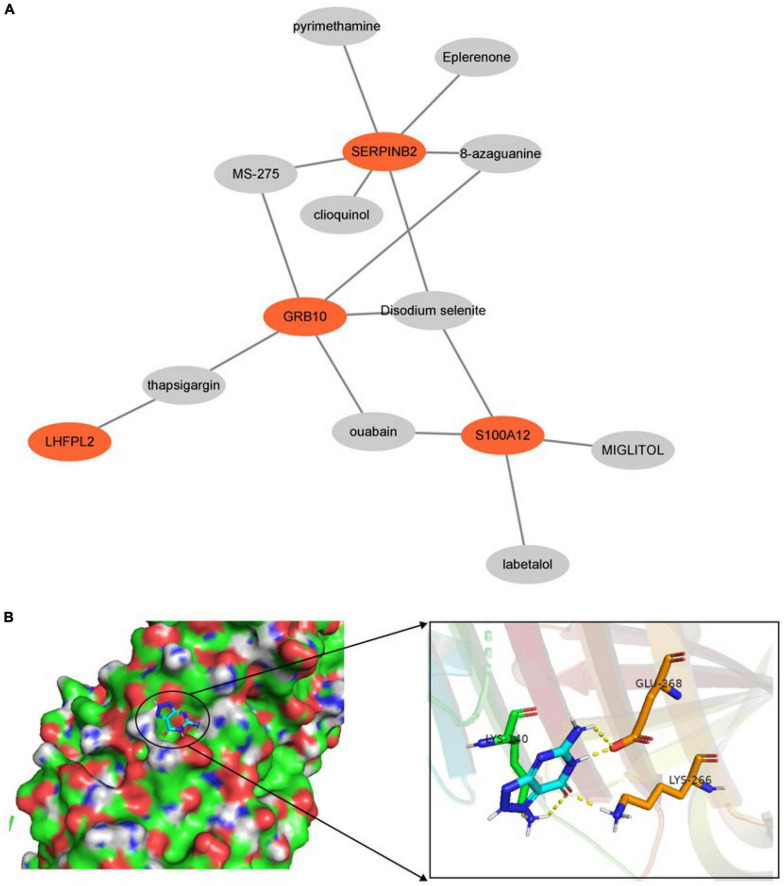
**(A)** Drug-biomarker network. **(B)** Display of molecular docking results.

Prediction of drugs with relevant effects was based on five potential biomarkers in peripheral serum of depression patients with the help of the DSigDB database. We considered the top 10 drugs in terms of *P*-value in the prediction results as potential drugs with biomarker interactions, including disodium selenite, MIGLITOL, ouabain, and thapsigargin (Figure 12). Furthermore, we also constructed drug-corresponding biomarker networks (Figure 13A) and selected the biomarker (SERPINB2) that is closely associated with the drug with the smallest *P*-value of the associated drug (8-azaguanine) as a representative and performed molecular docking through the DockThor online platform to verify the binding level between the biomarker and the drug, and visualized the results through PyMOL software. The results showed that the docking energy was –6.258 KJ/mol and displayed a good binding ability between biomarkers and drugs (Figure 13B).

### Biomarker clinical diagnostic capability

**FIGURE 14:**
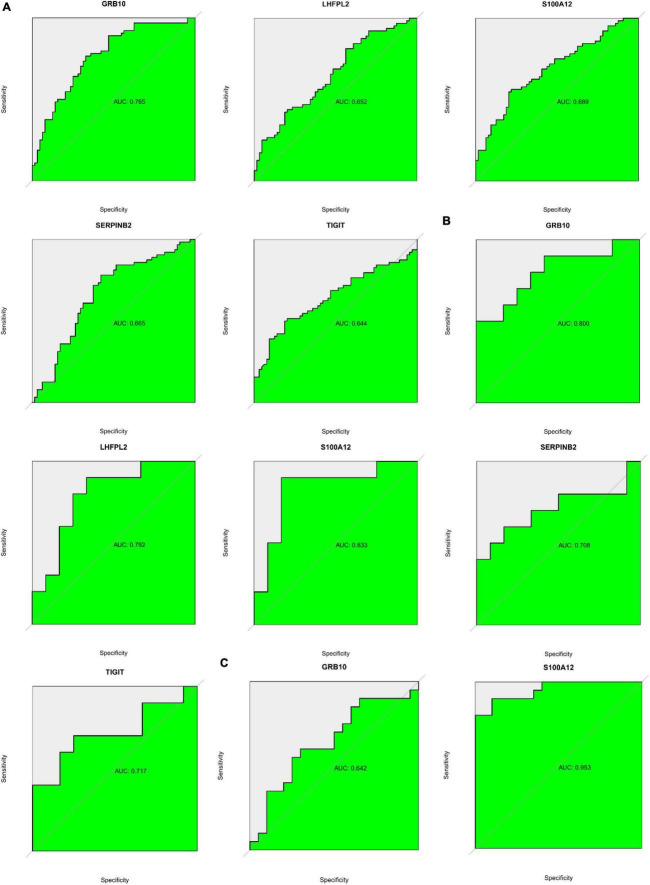
**(A)** ROC curve showing potential biomarkers in the GSE98793 dataset. **(B)** ROC curve showing potential biomarkers in the GSE76826 dataset. **(C)** ROC curve showing potential biomarkers in the GSE201332 dataset.

ROC analysis based on both GSE98793 and GSE76826 datasets suggested that S100A12, TIGIT, SERPINB2, GRB10, and LHFPL2 in the peripheral serum have the potential to act as diagnostic biomarkers for depression (Figures 14A,B). Among them, S100A12 and GRB10 both showed outstanding clinical diagnostic ability. Subsequently, ROC analysis of the GSE22132 dataset revealed that S100A12 may be the most potential diagnostic marker in peripheral blood for depression (Figure 14C).

### Mfuzz expression pattern clustering and functional analysis of optimal diagnostic biomarkers

**FIGURE 15:**
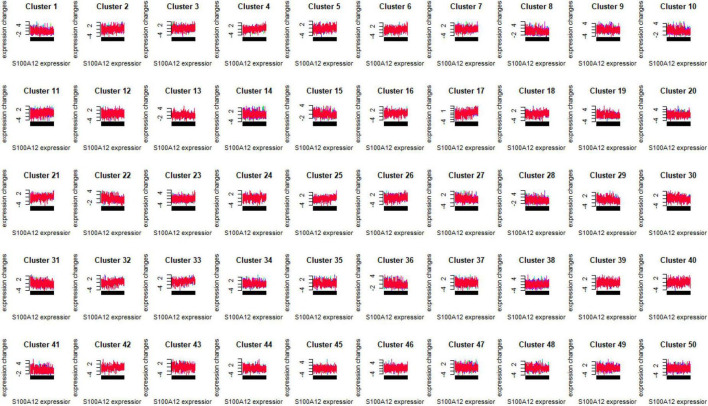
Mfuzz expression pattern clustering results.

**FIGURE 16:**
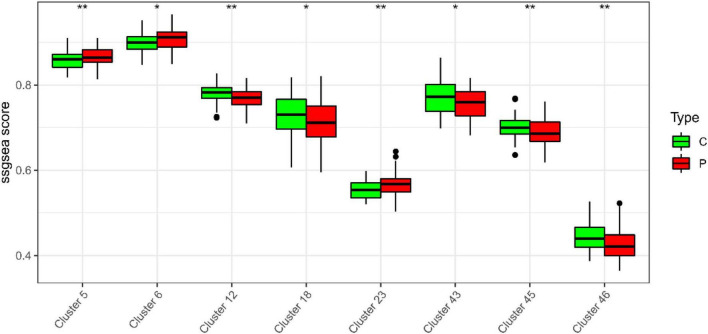
ssGSEA score of clustering module and expression characteristics between depression group (C) and normal group (P).

**FIGURE 17:**
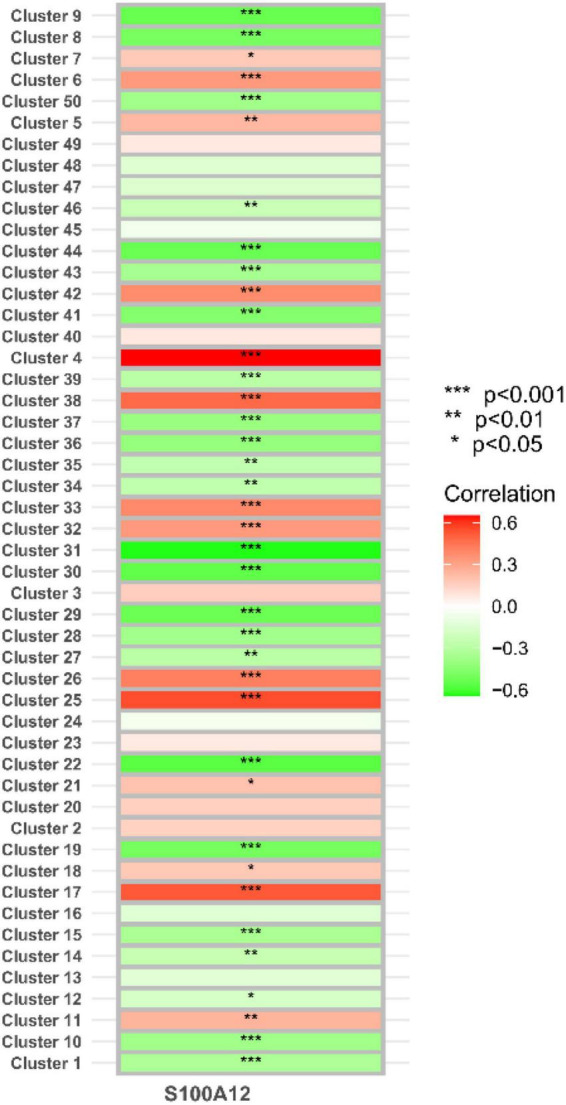
Correlation between clustering module and S100A12.

In the study, we clustered the Mfuzz expression pattern based on the expression level of S100A12, and obtained 50 clustering results (Figure 15), combined with the ssGSEA score of the clustering module and the expression characteristics, clustering between the depression group and the normal group (Figure 16). Correlation between class modules and S100A12 (Figure 17), Cluster6 was found to be the most closely linked gene module with S100A12.

**FIGURE 18:**
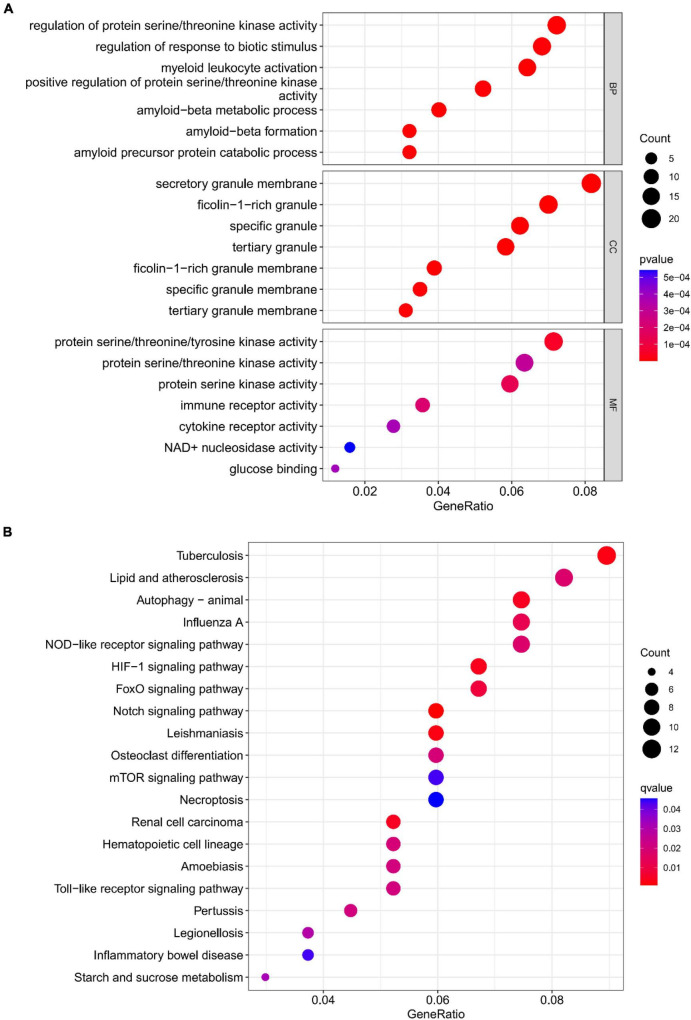
**(A)** Top 7 BPs, CCs, MFs, **(B)** top 20 KEGG pathways.

**FIGURE 19:**
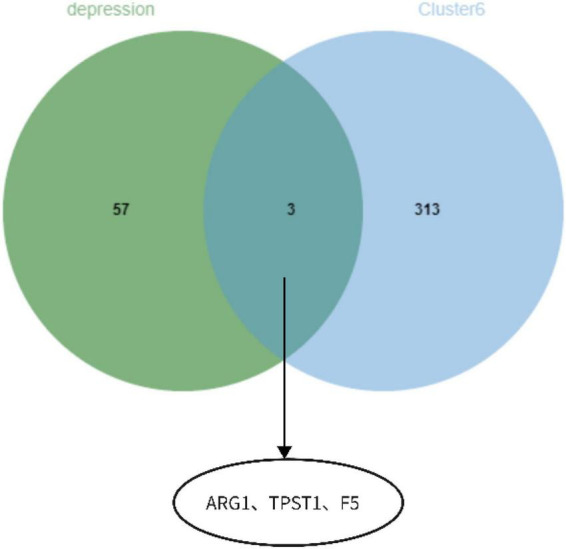
ARG1, TPST1, and F5 as core genes related to S100A12 expression in depression.

The functional enrichment results of Cluster6 module genes showed that these genes are involved in the regulation of immune receptor activity and are related to diseases such as atherosclerosis, which is consistent with the enrichment function of potential targets of depression. In addition, FoxO signaling pathway and mTOR signaling pathway were also found, which may help to further understand the pathological mechanism of depression (Figure 18). Finally, we identified ARG1, TPST1 and F5 as core genes associated with S100A12 expression in depression (Figure 19).

## Discussion

Depression, a prevailing global mental illness, imposes a heavy economic burden on patients and society. It is also a common co-morbid disorder that occurs concurrently with coronary heart disease, Parkinson’s disease, and anxiety disorders (11–13). Previous studies performed depression hub genes and functional analyses by WGCNA or differential analysis. This study is the first to combine WGCNA and differential analysis to screen hub genes, integrate machine learning algorithms for biomarker screening, and perform functional analysis and regulatory mechanism prediction on the obtained biomarkers (14, 15). The identification of these biomarkers will help in diagnosing diseases, selecting therapeutic approaches, and predicting therapeutic responses.

In this study, 550 DEGs were first detected in the peripheral blood of depressed individuals. Subsequently, we carried out a GSEA functional analysis of these DEGs and observed that these genes were present in pathways such as anti-folic acid resistance, ribosomes, and ribosome biogenesis in eukaryotes. Folic acid contributes to the proliferation and differentiation of hippocampal neural stem cells, and hippocampal neurons undergo plasticity changes in the case of folic acid deficiency, leading to memory and cognitive dysfunction (16). Normal ribosome synthesis contributes to cell survival, growth, and proliferation. Abnormal ribosomal protein expression or transcription is mostly present in depression patients as well as animal models of depression, and this ribosomal synthesis dysfunction may lead to neurogenic dysfunction and neuronal loss (17). WGCNA yielded 1,194 genes, and intersection analysis yielded 140 intersection genes. The functional analysis of these genes indicated that these targets were primarily associated with BPs such as antibacterial humoral responses, antibacterial peptide-mediated antibacterial humoral immune responses, humoral immune responses, and enriched in KEGG pathways such as *Staphylococcus aureus* infection and neutrophil outer trap formation. In addition, DO enrichment analysis identified key targets primarily involved in cardiovascular diseases such as atherosclerosis. Depression increases the risk of physical diseases, including cardiovascular disease, widespread bacterial infections, and autoimmune diseases, which is consistent with the results of our functional analysis (18). Inflammation is a protective immuno–vascular response that involves immune cells, blood vessels, and molecular mediators and is widely used to characterize immune-related processes *in vivo*; generally, external causes (e.g., microbial infections) or internal causes (e.g., atherosclerosis) can activate inflammatory responses to clear necrotic and damaged cells and initiate tissue repair (19). Immune dysregulation may have a crucial role in the pathological pathways of depression. An earlier study observed that patients in the depression group exhibited abnormal expression of pro-inflammatory and anti-inflammatory cytokines relative to those in the healthy group (20). Pro-inflammatory cytokines can inhibit the synthesis of 5-hydroxytryptamine in brain regions, affecting neuroplasticity and thus triggering depression (21). Furthermore, anti-inflammatory drugs may have a better antidepressant effect compared to placebo treatment.

Moreover, we investigated the correlation between 140 target genes and constructed PPI networks to obtain 60 hub genes. Subsequently, S100A12, TIGIT, SERPINB2, GRB10, and LHFPL2 in peripheral blood were identified as Potential diagnostic biomarkers in peripheral blood for depression by SVM–REF, Random forest, and LASSO algorithms. Among them, S100A12 is the most potential diagnostic biomarker. The functional analysis of gene modules related to S100A12 also indicated that it may be involved in the immune regulatory response in depression, and the core genes of ARG1, TPST1 and F5 related to its expression were found. S100A12, a member of the small calcium-binding S100 protein family, is secreted primarily by neutrophils, macrophages, and smooth muscle cells; it is released in inflammatory states and functions as a pro-inflammatory molecule and can be used as a diagnostic biomarker for various inflammatory diseases such as rheumatoid (22, 23). Co-inhibitory receptor (IR), a recently discovered molecule, protects the host from autoimmune responses and maintains peripheral self-tolerance; in addition, it maintains immune homeostasis, T-cell immunoglobulin, and ITIM structural domains (TIGIT) and plays a crucial role in immune regulation by promoting regulatory T cells (24). Serpin peptidase inhibitor, clade B (ovalbumin), member 2 (SERPINB2) is often expressed in lymphoid organs such as the spleen and in inflammatory responses or subjected to induced expression under various conditions of infection and pro-inflammatory stimuli. Studies on SERPINB2-knockout mice observed that macrophage-expressed SERPINB2 is involved in the regulation of adaptive Th1/Th2 immune responses (25, 26). Growth factor receptor-binding protein 10 (GRB10), a signaling adaptor protein encoded by imprinted genes, is involved in cell proliferation, neuronal development, and other processes. According to the expression data of GRB10 during neuronal development *in vitro*, this gene may be responsible for the regulation of neuronal function by suppressing the maternally expressed major promoter and activating the paternally expressed neuron-specific promoter (27). Fewer reports are available on lipoma HMGIC fusion partner-like 2 protein(LHFPL2) (28). No studies have reported a direct association of S100A12, TIGIT, SERPINB2, GRB10, and LHFPL2 with depression. However, based on previous studies, it is speculated that the above-mentioned biomarkers may be involved in the progression of depression through involvement in inflammatory responses or neuronal repair. Based on the results of the ROC (validation set AUC value > 0.7) analysis in the current study, S100A12, TIGIT, SERPINB2, GRB10, and LHFPL2 may possess potential as diagnostic biomarkers of depression.

The expression of 22 immune cells in depression and normal control groups was measured to understand the link between depression and the immune system. Blood samples from depression patients had increased expression levels of B cells naive, B cells memory, T cells CD8, and Mast cells resting The correlation of S100A12, TIGIT, SERPINB2, GRB10, and LHFPL2 with immune cells indicated that these genes were associated with the expression levels of macrophages MO, mast cells resting, macrophages M2, neutrophils, monocytes, B cells naive, T cells CD4 memory resting, and T cells CD8. A study involving lymphopenic mice confirmed that the adaptive immune system, consisting of T and B cells, serves as a potential factor in depression, as evidenced by a decrease in circulating T cells, regulatory B cells, and changes in the relative abundance of T cell subtypes in patients with depression (29). In addition, T cells may exert neuroprotective effects under stress and inflammation. For example, the generation of autoreactive T cells through CNS-specific antigen immunization reverses stress-induced reductions in hippocampal neurogenesis and depression-like behavior in rodents (30). B cells play a critical role in depression-related inflammatory responses by secreting the pro- and anti-inflammatory factors (31). Furthermore, mast cells distributed between ganglion cells and nerve fibers were activated by binding to IgE crosslink FcεRI high-affinity receptors to release a large number of mediators responsible for the production of inflammatory cytokines, thus exacerbating depression (32).

The biofunctional analysis of GSEA of S100A12, TIGIT, SERPINB2, GRB10, and LHFPL2 detected neuron generation-related pathways such as anti-folic acid resistance and ribosome biogenesis in eukaryotes, which were consistent with the results of our previous DEG functional analysis. In addition, we identified inflammation-related signaling pathways such as IL-17and TNF. The serum of depressed individuals has a higher expression of IL-17, which is a pro-inflammatory cytokine. This increased IL-17 expression is involved in the immune response and neuroimmune toxicity during depression pathology (33). IL-17 promotes the production of nitric oxide, which disrupts the blood-brain barrier and thus damages the brain; in addition, IL-17 induces structural remodeling of microglia and enhances the release of pro-inflammatory cytokines from microglia (34). The tumor necrosis factor (TNF), a central component of the innate type of immune system, is one of the most common cytokines studied in depression (35). During the progression of depression, TNF impairs the synthesis of monoamine neurotransmitters and the increased accumulation of neurotoxic metabolites, thus reducing the availability of 5-hydroxytryptamine synthesis (36). In addition, we explored the regulatory mechanisms of these five potential biomarkers. S100A12, TIGIT, SERPINB2, and GRB10 may be regulated by has-mir-164a-5p. However, few reports are available on their action mechanisms, which require further investigations in future studies. Furthermore, antidepressants such as disodium selenite and eplerenone were predicted. Disodium selenite is derived from selenium, and its ability to modulate the activity of selenized enzymes can achieve neuroprotective effects. Several neuropsychiatric disorders, including depression, are associated with selenium deficiency (37). Eplerenone is a type of mineralocorticoid-receptor (MR) drug. Research on the behavior of diabetes combined with depression revealed that eplerenone reduces neuroinflammation, promotes the maturation of brain-derived neurotrophic factor (BDNF), and exerts neuroprotective and antidepressant effects (38).

There are also some limitations in this study. First, the available transcriptome datasets in peripheral blood of depression are limited. In this study, we only used 1 training set and 2 validation set. Second, the biomarkers we obtained lack more literature support and validation of their expression characteristics in animal or clinical trials.

## Conclusion

To sum up, we screened 140 key targets in the peripheral serum of patients with depression. S100A12, TIGIT, SERPINB2, GRB10, and LHFPL2 identified as potential diagnostic biomarkers in peripheral blood of patients with depression using three machine learning algorithms. Among them, S100A12 is the most valuable diagnostic biomarker. The key targets in depression and the functional enrichment of 5 biomarkers all suggest changes in the immune system in depression, and more and more studies have begun to focus on the changes in the levels of inflammatory factors in patients with depression. The immune infiltration analysis also found immune cells closely related to depression. Depression has complex pathogenesis and heterogeneity, which brings great difficulties to the diagnosis and treatment of depression. The results of this study showed that the occurrence of depression is closely related to the immune system. Therefore, in future research, by studying the changing characteristics of the human immune system, it may provide new ideas for the prevention, diagnosis and treatment of depression. In addition, antidepressants such as disodium selenite and eplerenone were predicted. Collectively, our findings help in improving the diagnosis and treatment of depression. However, the action mechanisms of these five genes in the onset and progression of depression need to be further explored.

## Data availability statement

The datasets presented in this study can be found in online repositories. The names of the repository/repositories and accession number(s) can be found in the article/supplementary material.

## Author contributions

ZW conceived and designed the study. ZW, CC, and ZM collected and analyzed the data. ZW and CC wrote the manuscript. All authors contributed to the article and approved the submitted version.

## Footnotes

1
https://www.ncbi.nlm.nih.gov/gds

2
https://cn.string-db.org/

3
https://www.genecards.org/

4
https://www.networkanalyst.ca/

5
https://maayanlab.cloud/Enrichr/

6
https://pubchem.ncbi.nlm.nih.gov/
